# Pregnancy and Neonatal Outcomes With Levothyroxine Treatment in Women With Subclinical Hypothyroidism Based on New Diagnostic Criteria: A Systematic Review and Meta-Analysis

**DOI:** 10.3389/fendo.2021.797423

**Published:** 2021-12-10

**Authors:** Zheng Ding, Yindi Liu, Spyridoula Maraka, Nadia Abdelouahab, He-Feng Huang, William D. Fraser, Jianxia Fan

**Affiliations:** ^1^ The International Peace Maternity and Child Health Hospital, School of Medicine, Shanghai Jiao Tong University, Shanghai, China; ^2^ School of Medicine, Shanghai Jiao Tong University, Shanghai, China; ^3^ Division of Endocrinology and Metabolism, University of Arkansas for Medical Sciences, Little Rock, AR, United States; ^4^ Department of Medicine, Central Arkansas Veterans Healthcare System, Little Rock, AR, United States; ^5^ Knowledge and Evaluation Research Unit in Endocrinology, Mayo Clinic, Rochester, MN, United States; ^6^ Centre of Research and Central Hospital, University of Sherbrooke (CRCHUS), Sherbrooke, QC, Canada; ^7^ Faculty of Medicine and Health Sciences, University of Sherbrooke, Sherbrooke, QC, Canada

**Keywords:** subclinical hypothyroidism, pregnancy outcomes, neonatal outcomes, levothyroxine treatment, pregnancy

## Abstract

**Background:**

Subclinical hypothyroidism (SCH) during pregnancy has been associated with multiple adverse maternal and neonatal outcomes. However, the potential benefits of levothyroxine (LT4) supplementation remain controversial. Variations across studies in diagnostic criteria for SCH may, in part, explain the divergent findings on the subject. This study aimed to assess the effect of LT4 treatment on pregnancy and neonatal outcomes among pregnant women who were diagnosed as SCH based on the most recent diagnostic criteria.

**Methods:**

We conducted a systematic review and meta-analysis of the literature published from inception to January 2020. The search strategy targeted the studies on pregnancy and neonatal outcomes following LT4 treatment in women with SCH based on 2017 American Thyroid Association diagnostic criteria. Pooled effect sizes were estimated using fixed and random effect models, according to the absence or presence of heterogeneity which was assessed using the I-squared statistic. Sources of heterogeneity and the stability of results were evaluated through sensitivity analysis.

**Results:**

Of the 2781 identified references, 306 full-text articles were screened for eligibility. Finally, 6 studies including a total of 7955 participants were retained for analysis. Summary effect estimates indicated that pregnant women with SCH treated with LT4 had a lower risk of pregnancy loss [odds ratio (OR) = 0.55, 95% confidence interval (CI): 0.43-0.71], preterm birth (OR=0.63, 95% CI: 0.41-0.98) and gestational hypertension (OR = 0.78, 95% CI: 0.63-0.97) than those in control group.

**Conclusion:**

LT4 treatment in pregnant women with SCH may reduce the risk of pregnancy loss, preterm delivery and gestational hypertension.

## Introduction

Subclinical hypothyroidism (SCH) is defined as the presence of an elevated thyroid stimulating hormone (TSH) concentration with a normal serum free thyroxine (FT4) level. It is the most common thyroid dysfunction during pregnancy, with prevalence ranging from 3.5% to 14% depending on the different cutoff values for TSH, ethnicity and iodine consumption (1, 2). Several studies have reported on the association between SCH during pregnancy and the risk of adverse pregnancy and neonatal outcomes, including miscarriage, premature birth, preeclampsia, gestational diabetes, gestational hypertension, premature rupture of membranes, intrauterine growth restriction, and low birth weight (3–8).

Some retrospective or RCT studies have reported that levothyroxine (LT4) supplementation can significantly decrease the risk of either pregnancy loss (9) or preterm birth (10). Another retrospective study comparing pregnant women with SCH treated with LT4 to women not receiving the medication also suggested a possible beneficial effect of LT4 on pregnancy loss (11). Some studies observed no effect on pregnancy loss or preterm birth (12), or the studies yielded inconclusive results (13). One possible explanation for these divergent findings between studies is the un-uniformity of TSH cutoff values used for the diagnosis of SCH. It should be noted that diagnostic criteria for SCH in pregnancy have evolved over time.

The new 2017 American Thyroid Association (ATA) guideline advocates the use of population-based reference ranges of TSH during pregnancy, whereas if these ranges are not available, they recommended using 4.0mIU/L as the upper reference limit of TSH for the first trimester, that was higher than the 2.5mIU/L cutoff which was recommend in the 2011 ATA guideline. In the new 2017 ATA guideline (14), LT4 therapy is strongly recommended for thyroid peroxidase antibody-positive (TPOAb+) women with a TSH greater than the pregnancy-specific reference range (or TSH >4.0mIU/L), whereas LT4 therapy may be considered for TPOAb+ women with TSH >2.5mIU/L and TSH<4.0mIU/L, or thyroid peroxidase antibody-negative (TPOAb-) women with TSH >4.0mIU/L and <10.0mIU/L. The latter are, however, weak recommendations based on low to moderate quality evidence.

On the other hand, a lack of consensus concerning optimal treatment for SCH in pregnancy is reflected in a variety of clinical practice guidelines, including those of the European Thyroid Association (ETA) (15) and the Endocrine Society (ES) (16). In the 2014 ETA guideline, the upper reference limits of TSH used for the diagnosis of SCH are recommended as 2.5 mU/l, 3.0 mU/l and 3.5 mU/l for 1st, 2nd and 3rd trimester, if trimester-specific reference ranges of TSH are not available. The 2014 ETA guideline recommends that women with SCH should take LT4 to ensure a TSH level of <2.5 mIU/l, regardless of TPOAb status. And the ES guideline recommends that all women with a TSH > 2.5 mIU/L and normal FT4 in the first trimester should be treated with LT4, irrespective of their TPOAb status.

Before the 2017 ATA guideline, the recommended TSH diagnostic cutoff value for SCH during pregnancy was 2.5mIU/L in the first trimester. Although there were previously published studies on the association between LT4 treatment for SCH and the pregnancy outcomes, the results remained controversial due to the use of different TSH diagnostic cutoff values for SCH, e.g.,>2.5mIU/L or different local pregnancy-specific reference ranges. A recent meta-analysis by Nazarpour et al. (17) evaluated the benefits of LT4 treatment on pregnant women with SCH. However, as regards the TSH cutoff values, that ranged from 2.5mIU/L to 4.5mIU/L in the included studies. In our previous observational study (18), we analyzed the relationship between TSH and FT4 among 46,262 pregnant women. FT4 was relatively constant when serum TSH levels were between 0.5 to 4.0mIU/L, with a median value of 14.0 pmol/L to 15.0 pmol/L. However, FT4 levels began to decrease significantly when TSH levels were above 4.0mIU/L. Out data suggest that the benefits of LT4 supplementation may not be obvious for pregnant women when the diagnostic cutoff of TSH is less than 4.0mIU/L.

The variations in the results between studies are not conducive to the production of clinical practice guidelines. Therefore, it is important to review the evidence on the association of LT4 supplementation with the risk of adverse pregnancy outcomes in women with SCH based on the new 2017 ATA diagnostic criteria. Thus, we performed a systematic review to investigate the effects of LT4 treatment on pregnancy and neonatal outcomes, with the goal of validating the current ATA guidelines and providing useful information for the clinical management of these patients. Our study based on the unified diagnostic criteria of the new 2017 ATA guideline should be helpful in clinical practice.

## Methods

### Literature Search Strategy

This systematic review and meta-analysis was performed according to the study protocol registered in the PROSPERO database (CRD42020168962). We followed the preferred reporting items for systematic reviews and meta-analyses described in the PRISMA statement (19). A comprehensive literature search regarding the effects of LT4 treatment on pregnancy outcomes in women with SCH was conducted to identify all related studies published from inception to January 2020 without language restrictions. The databases that were searched included PubMed, Embase, Web of Science, Cochrane Controlled Trials Register and CNKI (China National Knowledge Infrastructure). The search strategy targeted human studies. We also reviewed the relevant studies in references by conducting manual searches when necessary. According to titles, abstracts and keywords, the studies were screened independently by two reviewers (Ding Z and Liu YD). After deleting duplicates, full-text articles of all eligible studies were obtained for independent review by two authors in duplicate. Any disagreements between reviewers regarding inclusion were settled by consensus or by consultation with a third reviewer (W Fraser).

The following terms were used for the search: [subclinical (Title/Abstract)] OR [sub-clinical(Title/Abstract)] AND [“Hypothyroidism”(Mesh)] OR [hypothyr*(Title/Abstract)] OR [hypo-thyr*(Title/Abstract)] OR [thyroid deficien*(Title/Abstract)] OR [thyroid insufficien*(Title/Abstract)] AND [“Pregnancy”(Mesh)] OR [“Pregnancy Outcome”(Mesh)] OR [pregnancy outcomes(Title/Abstract)] OR [pregnan*(Title/Abstract)] OR [pregnancy outcome(Title/Abstract)] AND [“Thyroxine”(Mesh)] OR [levothyroxine(Title/Abstract)] OR [LT4(Title/Abstract)] OR [thyroxine supplementation(Title/Abstract)] OR [thyroxine(Title/Abstract)] OR [synthroid(Title/Abstract)].

### Eligibility Criteria

To assess the effect of LT4 treatment on pregnant women with SCH compared to those who did not receive LT4, all relevant RCTs or cohort studies were sought for possible inclusion in the meta-analysis.

Studies were eligible if: (1) they compared pregnancy outcomes between those with LT4 supplementation and placebo or no treatment; (2) studies included women who were diagnosed with SCH in pregnancy, based on the new 2017 ATA criteria: TSH level above the upper limit of pregnancy-specific reference range or (if unavailable) more than 4.0mIU/L and less than 10.0mIU/L; (3) data on maternal and/or neonatal outcomes were reported or could be obtained by personal communication. Studies were excluded if: (1) they were case series or case-control studies; (2) the study did not provide standard design methods, such as inappropriate grouping or irrelevant diagnostic criteria; (3) there was a lack of sufficient data in the published study on outcomes of interest.

### Data Extraction

Two reviewers independently extracted relevant study information in duplicate using standardized data extraction forms, and any disagreements between the reviewers were resolved by discussion with the research team. The following information was extracted from each eligible study: (1) first author(s), year of publication, country, study design, sample size; (2) baseline summary statistics on the study population characteristics including age, body mass index(BMI), race/ethnicity; (3) the specific values of TSH; (4) numbers of pregnant women with SCH who were treated versus untreated with LT4, gestational age at LT4 initiation and the dosage of LT4 treatment; (5) numbers of patients with thyroid antibody status (positive and negative) in intervention and control groups; (6) maternal and neonatal outcomes. In addition, the information about iodine status, history of thyroid disease, and safety outcomes of LT4 treatment also was extracted if they were available. The corresponding author or first author was contacted for more information if the data presented in the article were insufficient for analyses. Both reviewers performed a quality control check between the final data included in the meta-analysis and the original publications.

### Outcome Measures

We extracted data for: (1) pregnancy loss (miscarriage, fetal death and stillbirth), (2) preterm birth or preterm delivery, (3) maternal complications (preeclampsia, gestational hypertension, gestational diabetes, placental abruption or placenta previa), and (4) neonatal complications[fetal growth restriction, small for gestational age, low birth weight, low Apgar score, fetal distress, neonatal intensive care (NICU) admission, neonatal death, congenital malformation].

### Quality Assessment of the Included Studies

Quality assessment of included studies was conducted independently by two reviewers. For the cohort studies, we used the Newcastle-Ottawa Quality Assessment Scale (NOS) (20) which has a range of 0 to 9 points based on three aspects, including participant selection, comparability of the study groups and ascertainment of the outcomes. Scores of 3 or less suggest low quality, scores between 4 and 6 suggest a medium level of quality, and a score of 7-9 suggests a high level of quality.

For the RCTs, we used the Jadad scale (21), which is based on four criteria: random sequence generation, randomization concealment, blinding, and dropout or withdrawal. The score ranges from 0 to 5 and a total score of ≤ 3 or > 3 indicates low or high quality of reporting, respectively.

### Risk of Bias Assessment

For the RCTs, the Cochrane risk of bias tool was used (22), which determines risk of bias based on including random sequence generation, allocation concealment, blinding of participants and personnel, blinding of outcome assessment, incomplete outcome data, and selective reporting. Inconsistencies were resolved by consensus between the reviewers.

For cohort studies, eight domains of risk of bias were assessed, including selection of exposed and non-exposed cohorts, similarity of co-interventions between groups, the assessment of exposure, presence of the outcome of interest at start of study, assessment of the presence or absence of prognostic factors, adjustment of the statistical analysis for prognostic analysis, the assessment of outcome, and adequacy of the follow-up of cohorts.

The tool provides a summary measure of bias for each study categorized as “low risk of bias”, “high risk of bias” or “unclear risk of bias”.

### Statistical Analysis

The R3.6.0 software package was used to conduct statistical analyses. A standard meta-analysis approach was performed to compare the included studies and evaluate the pooled odds ratios (ORs) of outcomes. The OR was used to describe the effect size, and the corresponding 95% confidence interval (95% CI) was used to express the pooled results. Forest plots were used to graphically represent the weighted ORs of outcomes. Heterogeneity was evaluated by the I-squared (*I* ^2^) index, the values over 50% interpreted as heterogeneity. A fixed effects model was used when *I* ^2^< 50%, otherwise, the random effects model was used. Inter-study variance was evaluated by calculating Tau^2^, which represents the estimated standard deviation of the underlying effects on studies. Sensitivity analyses were conducted by eliminating a study to verify the potential influence on the pooled effect, to search for sources of heterogeneity and to evaluate whether the results of meta-analysis were stable and reliable. Publication bias was not assessed due to the limited number of publications.

### Subgroup and Sensitivity Analyses

We performed subgroup analyses to further analyze the effects of LT4 supplementation on pregnancy outcomes based on the TPOAb status of the study participants (positive or negative) and study design (RCT or cohort study). The sensitivity analyses, which was conducted by removing each study in turn and estimating the overall effect of the remaining studies sequentially, were conducted to assess the effect of each individual study and the stability of the results of the meta-analysis.

## Results

### Literature Search Results and Study Selection

**Figure 1:**
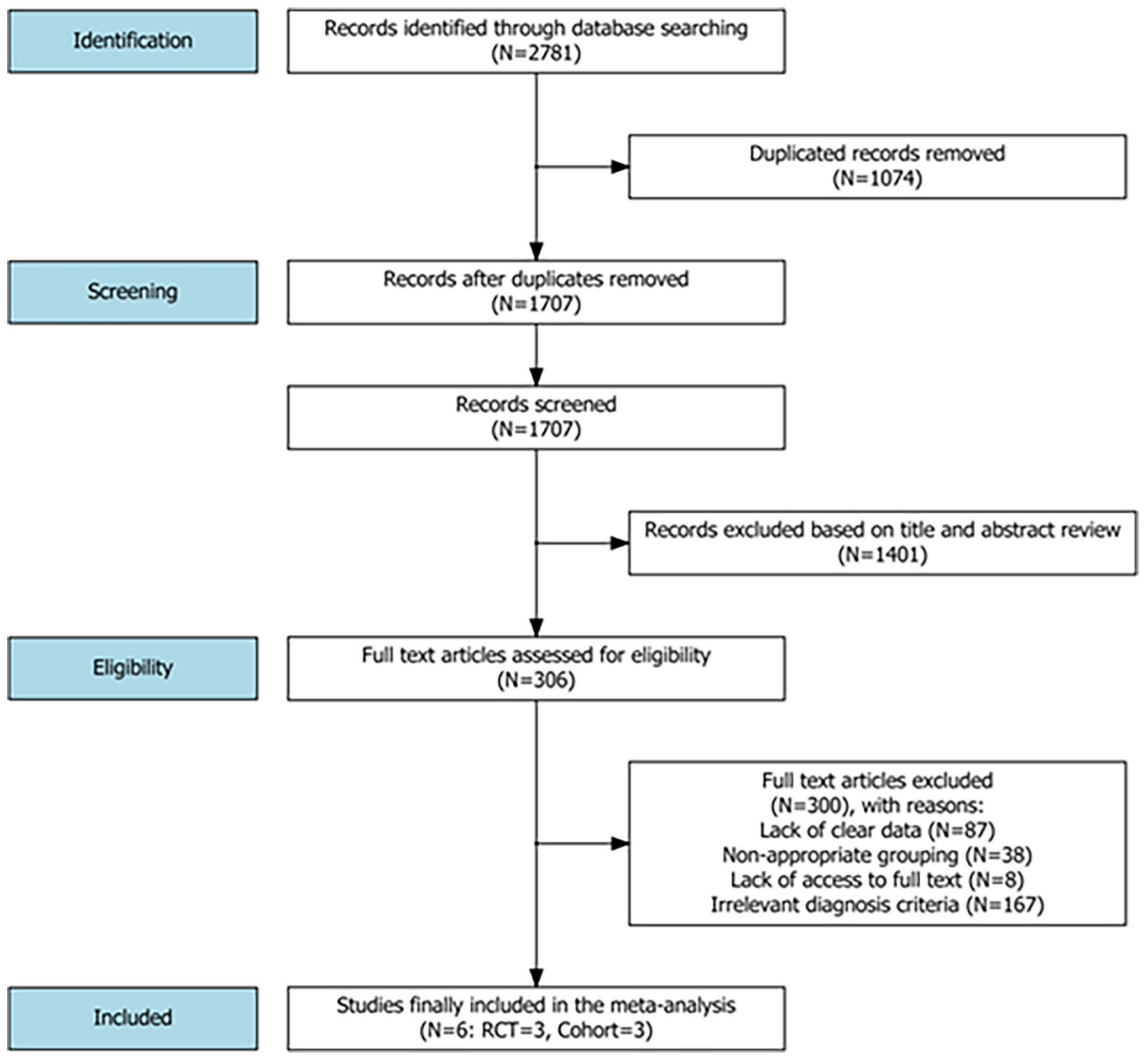
A flowchart of the literature search strategy and study selection.

A flowchart of the literature search and its results is illustrated in **Figure 1**. Overall, the literature search initially yielded 2781 articles from the PubMed, Embase, Web of Science, Cochrane and CNKI databases. After excluding duplicate studies and reviewing the titles and abstracts from the identified references, 306 full-text articles were assessed for eligibility. Finally, 6 studies including a total of 7955 patients met the eligibility criteria and were included in the meta-analysis (10, 23–27), one of which was retained after obtaining relevant additional data from the first author of the study (25).

### Characteristics of Included Studies

In all, six studies published between 2015 and 2019 [three RCTs (10, 23, 27) and three cohort studies (24–26)] were eligible for the meta-analysis. These studies were conducted in the USA (23, 25), China (24, 26) and Iran (10, 27). In these studies, for women in the intervention group, LT4 was initiated in pregnancy and maintained until delivery. The gestational age at initiation of LT4 treatment varied across studies. Adjusted dosages of LT4 were used in three studies depending on the individual TSH level (23, 24, 27), whereas a fixed dosage of 1μg/kg/day was administered in one study (10). In the two remaining studies, the dosages were not described in detail (25, 26). The iodine status was provided in two included studies (10, 23). The information about history of thyroid disease and safety outcomes of LT4 treatment was provided in only one study (25). All included studies reported data on pregnancy or neonatal outcomes. Study characteristics are summarized in **Table 1**.

**Table 1:** Characteristics of the included studies.

Author	Year	Country	Study design	Gestational age at LT4 initiation	Group size	
					Intervention	Placebo
Ye W et al. (24)	2019	China	Cohort study	<14 weeks	171	112
Nazarpour S et al. (10)	2018	Iran	RCT	11.4±4 weeks	87	60
Maraka S et al. (25)	2017	USA	Cohort study	NA	513	697
Casey BM et al. (23)	2017	USA	RCT	8-20 weeks	339	338
Nazarpour S et al. (27)	2017	Iran	RCT	10.8±4 weeks	38	34
Yang J et al. (26)	2015	China	Cohort study	<12 weeks	1236	806
**Author**	**Year**	**SCH definition**		**Outcomes**		**Quality**
Ye W et al. (24)	2019	TSH >4.0 mIU/L		Miscarriage, Gestational diabetes, Gestational hypertension, Preterm birth, Placental abruption, FGR, LGA		High
Nazarpour S et al. (10)	2018	TSH >4.0 mIU/L		Preterm delivery, Placental abruption, Stillbirth, Neonatal admission		High
Maraka S et al. (25)	2017	TSH 4.10-10.0mIU/L		Pregnancy loss, Preterm delivery, Preterm labor, PROM, Placental abruption, Gestational diabetes, Gestational hypertension, Preeclampsia, Poor fetal growth		High
Casey BM et al. (23)	2017	TSH >4.0 mIU/L		Preterm birth, Placental abruption, Gestational hypertension, Preeclampsia, Gestational diabetes, Stillbirth or miscarriage, Neonatal death, Admission to NICU, Birth weight <10%, Respiratory distress syndrome, Child IQ		High
Nazarpour S et al. (27)	2017	TSH >4.0 mIU/L		Preterm delivery, Neonatal admission, Miscarriage, Placental abruption, Stillbirth, Neonatal TSH levels		High
Yang J et al. (26)	2015	TSH 5.22-10.0mIU/L		Miscarriages, Preterm birth, Gestational hypertension, Gestational diabetes, FGR, LGA		High

RCT, Randomized Controlled Trial; SCH, Subclinical Hypothyroidism; TSH, Thyroid Stimulating Hormone; FGR, Fetal Growth Restriction; LGA, Large for Gestational Age; PROM, Premature Rupture of Membrane; NICU, Neonatal Intensive Care Unit; IQ, Intelligence Quotient.

### Quality Assessment and Risk of Bias Assessment

The quality of the three included retrospective cohort studies was high, with two NOS scores assessed as 7 (24, 26) and one as 8 (25), and the quality of the three RCTs was also high, with one Jadad score assessed as 5 (10) and the other two as 4 (23, 27). The high quality of RCT was based on their randomization schemes, the use of randomization hiding, participant blinding, and low levels of loss to follow-up.

The three retrospective cohort studies indicated a low risk of bias. For three RCT studies, the risk of bias was low mainly based on the random sequence generation, blinding of outcome assessment, minimal incomplete outcome data and absence of selective reporting. The results of quality assessment and risk of bias assessment were illustrated in **Supplementary Figure S1**.

### Effects of LT4 Supplementation on Pregnancy and Neonatal Outcomes

**Figure 2:**
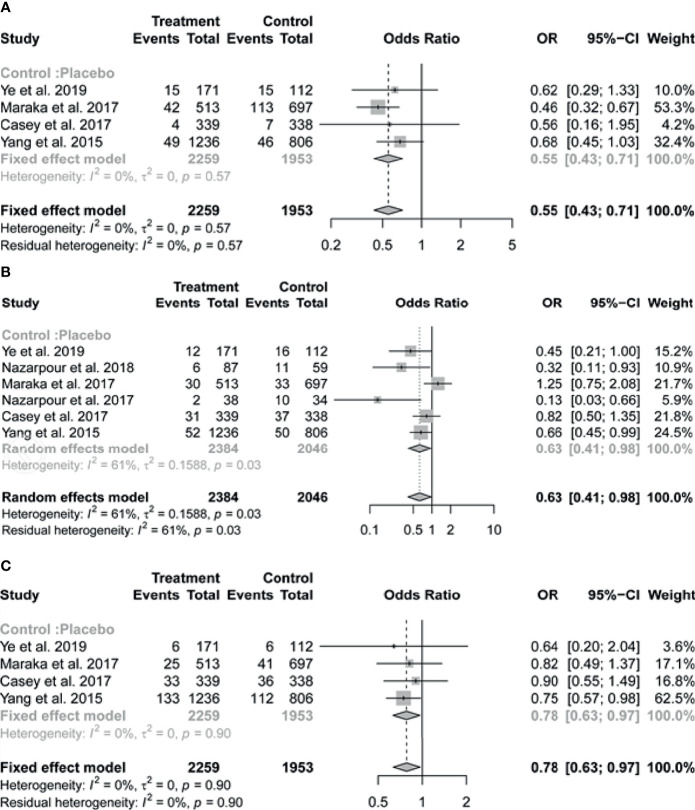
Forest plots of odds ratio (OR) and 95% confidence interval (CI) of pooled studies comparing levothyroxine treatment group to control group for the risk of **(A)** pregnancy loss, **(B)** preterm birth and **(C)** gestational hypertension.

Of the six studies included in the review, four reported on the outcome of pregnancy loss. The pooled results indicated that pregnant women with SCH who received treatment with LT4 had a substantially lower risk of pregnancy loss (OR = 0.55, 95% CI: 0.43-0.71, *I* ^2^ = 0%) compared to those in the untreated group (**Figure 2A**).

**Figure 3:**
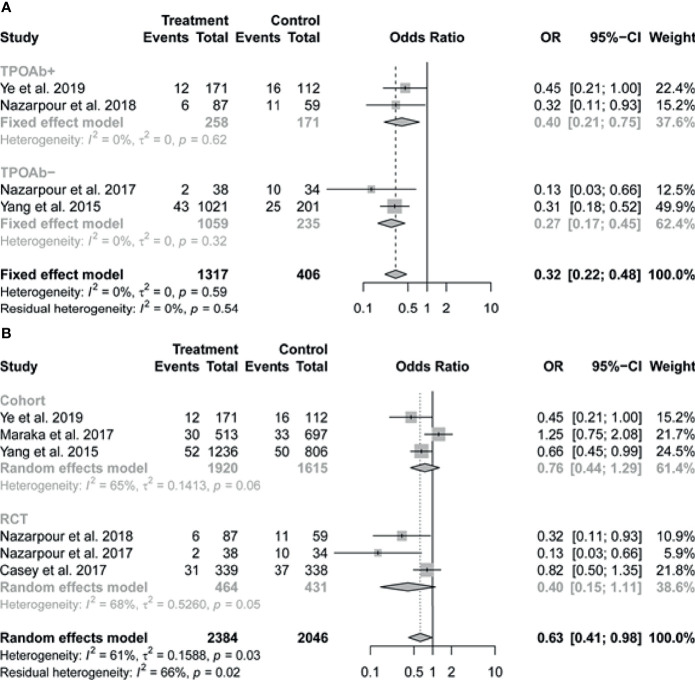
Forest plots of pooled results on the association between levothyroxine treatment and the risk of preterm birth by subgroup analysis based on **(A)** TPOAb status (positive or negative) and **(B)** study design (RCT or cohort study). TPOAb, Thyroid peroxidase antibody.

The pooled data of the six studies reporting on preterm birth in patients with SCH showed that LT4 therapy was associated with significant reduction in the risk of preterm birth (OR 0.63, 95% CI 0.41-0.98; *I* ^2^ = 61%) (**Figure 2B**). In subgroup analysis, there was no evidence of interaction of antibody status in the association between LT4 treatment and preterm birth, the pooled results by TPOAb status (positive or negative) were consistent with the overall results, with I^2^ = 0% (**Figure 3A**). We further performed the subgroup analysis based on the study design (RCT or cohort study), the pooled results suggested that relatively high weight of certain study would influence the subgroup result or the overall result (**Figure 3B**).

Four of the six studies contributed to the meta-analysis on gestational hypertension. We found that LT4 supplementation significantly reduced the risk of gestational hypertension (OR = 0.78, 95% CI: 0.63-0.97, *I* ^2^ = 0%) (**Figure 2C**). Preeclampsia was reported only in the study of Casey et al. (intervention vs placebo: 22/339 vs 20/338).

We did not find statistical evidence of an association of LT4 therapy with other maternal or neonatal complications, including gestational diabetes (OR = 0.94, 95% CI: 0.58-1.51, *I* ^2^ = 73%), placental abruption (OR=0.73, 95% CI: 0.38-1.41, *I* ^2^ = 21%), fetal growth restriction (OR=0.95, 95% CI: 0.62-1.44, *I* ^2^ = 63%) or small for gestational age (OR=0.83, 95% CI: 0.64-1.07, *I* ^2^ = 34%) (**Supplementary Figure S2**).

There was no evidence of statistical heterogeneity across studies for the outcomes of pregnancy loss, gestational hypertension, placental abruption, or small for gestational age. However, with respect to the outcomes of preterm birth, gestational diabetes and fetal growth restriction, results indicate a moderate heterogeneity across studies.

### Sensitivity Analysis

The sensitivity analyses were conducted to assess the impact on the effect estimate and explain possible inconsistencies of results by excluding each study in turn. With regards to preterm birth, the pooled ORs of the remaining studies were observed to change after omitting each study one by one but no change was noted when removing Maraka et al., (25) or Casey et al., (23). With regards to pregnancy loss, after excluding individual studies that differed according to the TPOAb status, there were small changes in the pooled ORs but no change in the directionality of the effect and all the pooled ORs of the remaining studies were consistent with the overall effect. The results of the sensitivity analysis were illustrated in **Supplementary Figure S3**.

## Discussion

The results of several previous studies have indicated an increased risk of pregnancy complications associated with SCH during pregnancy. However, only a small number of studies have investigated the impact of LT4 treatment on adverse maternal and neonatal outcomes. The role of LT4 supplementation in pregnant women with SCH remains controversial.

Maraka et al. (11) suggested a possible beneficial effect among pregnant women with SCH who received LT4 compared to those who did not receive treatment. In their study, pregnant women with SCH, defined as a TSH >2.5 mIU/L for the 1st trimester or >3 mIU/L for the 2nd and 3rd trimesters but ≤10 mIU/L, were divided into two groups according to whether or not they received LT4. The results indicated that LT4 therapy for SCH was associated with a decreased risk of low birth weight (LBW) and low Apgar score, but no evidence of an effect on pregnancy loss. Nazarpour et al. (10, 27) reported that the risk of adverse pregnancy outcomes was related to TPOAb status. To explore the effects of LT4 treatment on pregnant women with autoimmune thyroid disease, they conducted a prospective study among pregnant women with TPOAb+ status. The results indicated that LT4 treatment from first trimester to delivery decreased the risk of preterm birth (27). Furthermore, Nazarpour et al. (10) evaluated the benefits of LT4 treatment on pregnancy outcomes in women with SCH and TPOAb- status. A single-blinded RCT was undertaken in pregnant women who had SCH and TPOAb- status. When using the TSH cutoff value of 2.5mIU/L, no significant difference was observed in the rate of preterm delivery between the group treated with LT4 and those who received no treatment. However, when using a threshold value of 4.0mIU/L, a significantly lower rate of preterm delivery was observed among women receiving LT4 compared to controls. However, other studies were unable to demonstrate a benefit of LT4 treatment on pregnancy loss or preterm birth among euthyroid pregnant women with TPOAb+ status (12, 28). In an intervention trial, Negro et al. (12) assessed the effect of LT4 therapy on the rate of miscarriage and preterm delivery in pregnant women with TSH<2.5 mIU/L and TPOAb+ status. The results showed that the LT4 intervention had no impact on the rate of miscarriage or preterm delivery in TPOAb+ women with a TSH level between 0.5 and 2.5 mIU/L.

Some variations in the results across studies may be attributed to the fact that there was no uniform cutoff value for TSH in the diagnosis of SCH. In 2011, the initial recommendation by the ATA was the use of a laboratory or population-based pregnancy-specific TSH reference range. If unavailable, a TSH upper limit of 2.5, 3.0 and 3.0 mIU/L in the 1st, 2nd and 3rd trimesters was suggested to be used, respectively (29). This guideline was revised in 2017, recommending a TSH cutoff value more than an upper reference limit of the pregnancy-specific TSH reference ranges or more than 4mIU/L in the first trimester if unavailable (14). Given the variations in conclusions across studies, we performed the present meta-analysis to investigate the effects of LT4 supplementation on pregnancy outcomes in women with SCH, based on the 2017 ATA new diagnostic criteria for SCH in pregnant women. In this systematic review and meta-analysis, we found evidence of beneficial effects of LT4 supplementation on the risk of pregnancy loss and preterm birth in women with SCH, as well as a reduction in the risk of gestational hypertension. Maraka et al. (30) reported that SCH during pregnancy is associated with multiple adverse maternal and neonatal outcomes, and the value of levothyroxine therapy in preventing these adverse outcomes remains uncertain. In their meta-analysis, SCH was defined as an elevated TSH concentration with normal serum T4 level or as an elevated TSH concentration between 2.5 and 5 mIU/L. In contrast, this meta-analysis was conducted using the new diagnostic criteria in the 2017 ATA guidelines.

In this meta-analysis, six studies were included in the analysis regarding the association between LT4 intervention and the rate of pregnancy loss or preterm birth. In order to determine whether the effect of LT4 therapy is mainly against SCH rather than TPOAb status, we performed a subgroup analysis. There was no evidence of interaction of antibody status in the association between LT4 treatment and preterm birth. The pooled results by TPOAb status (positive and negative) were consistent with the overall results. However, this consistency didn’t occur in other pregnancy outcomes. The differences indicate that the TPOAb status might be an effect modifier of the relationship of LT4 to pregnancy outcomes but not all. In sensitivity analysis, we excluded each individual study one by one, with regards to pregnancy loss, there were no significant change in the directionality of the effect and all the pooled ORs of the remaining studies were consistent with the overall effect. As regards the preterm birth, we also performed the subgroup analysis based on the study design (RCT or cohort study), the pooled results suggested that a high weight of certain study would influence the subgroup result or the overall result. In sensitivity analysis, the pooled ORs of the remaining studies were observed to change after omitting each study in turn but no change was noted when removing Maraka et al., 2017 or Casey et al., 2017. One of explanation is that these studies account for a relatively high weight in the results. Further studies are warranted to confirm the associations and elaborate the underlying molecular mechanisms.

The main strength of the current meta-analysis compared to others is that the included studies assess the effect of LT4 in pregnant women with SCH based on 2017 ATA new diagnostic criteria. Many previous studies on the effect of LT4 treatment in pregnant women with SCH used different cutoff values for TSH. However, several limitations of this study should be considered when interpreting the findings. First, a limited number of studies met the eligibility criteria. This led to a decrease in power when conducting subgroup analyses. We didn’t perform the subgroup analysis on pregnancy loss and gestational hypertension just due to the limited number of included studies. Second, the included studies consisted of RCTs and cohort studies. In the RCTs, the TPOAb status had been balanced between the intervention and control group, whereas in observational cohort studies, there was an imbalance among groups in antibody status (26), then this could be a confounding variable. So in the observational studies, there was the potential that the associations could be confounded by antibody status or other variables. More researches are needed to examine whether the effect of LT4 treatment depends on antibody status. Additionally, RCT studies are more likely to produce unbiased results, however, the number of RCT studies on this topic is still limited and the size of these studies is small. We believe that by combining clinical trials and observational studies in this analysis, we provide the totality of the strongest evidence to date on this subject. We also applied strict criteria regarding inclusion, which we believe strengthens the conclusions of our study. Further large RCT studies on this subject are still necessary. Third, the studies differed in terms of the LT4 dosage, with some studies using fixed dosages, while others titrated dose to achieve a target TSH level. Fourth, there may be publication bias in included studies due to the unpublished negative results of studies. Another limitation is that we could not assess the effects of other factors such as iodine status of the study populations, ethnicity, women’s BMIs, gestational age at the time of diagnosis of thyroid dysfunction or variations in the starting time of LT4 treatment. These confounders may cause bias with respect to pregnancy outcomes between the groups.

The results of the present meta-analysis support the recommendation that LT4 should be administered in pregnant women with SCH and TSH > 4.0mIU/L to reduce the risk of pregnancy loss, preterm birth and gestational hypertension. These results may help to optimize clinical decision-making strategies.

## Conclusion

To our knowledge, this study is the first meta-analysis that shows that LT4 supplementation is associated with a decreased risk of pregnancy loss, preterm birth, and gestational hypertension in women with SCH based on the new 2017 ATA diagnostic criteria. Considering the limited number of available studies included in this meta-analysis and the inevitable heterogeneity, the findings cannot be generalized to patients diagnosed with SCH based on other criteria. Further large RCTs may be warranted to further strengthen these conclusions.

## Data Availability Statement

The original contributions presented in the study are included in the article/**Supplementary Material**. Further inquiries can be directed to the corresponding authors.

## Author Contributions

ZD and YL screened and reviewed the included studies in duplicate. ZD wrote the original draft and YL analyzed the data. SM and NA revised and edited the manuscript. WF reviewed the manuscript. H-FH and JF designed the work. All authors contributed to the article and approved the submitted version.

## Funding

This work was supported by the National Key Research and Development Program of China (2018YFC1004600); National Natural Sciences Foundation of China (81661128010); and the Canadian Institutes of Health Research (HLT 151517).

## Conflict of Interest

The authors declare that the research was conducted in the absence of any commercial or financial relationships that could be construed as a potential conflict of interest.

## Publisher’s Note

All claims expressed in this article are solely those of the authors and do not necessarily represent those of their affiliated organizations, or those of the publisher, the editors and the reviewers. Any product that may be evaluated in this article, or claim that may be made by its manufacturer, is not guaranteed or endorsed by the publisher.
