# Supplementary material for: Pregnancy and Neonatal Outcomes With Levothyroxine Treatment in Women With Subclinical Hypothyroidism Based on New Diagnostic Criteria: A Systematic Review and Meta-Analysis
Source: Front Endocrinol (Lausanne). 2021 Dec 10;12:797423. doi: 10.3389/fendo.2021.797423 (PMC8703220; doi:10.3389/fendo.2021.797423)
Supplement: Supplementary file 1 [file DataSheet_1.docx]

**Supplementary Figure S1**: Quality assessment (A、B) and risk of bias assessment (C、D)


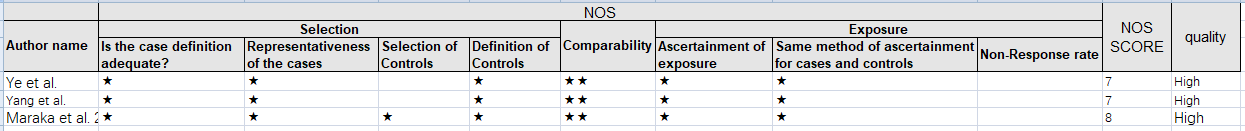


(A) Quality assessment-NOS for cohort study. Scores of 3 or less suggest low quality, scores between 4 and 6 suggest a medium level of quality, and a score of 7-9 suggests a high level of quality


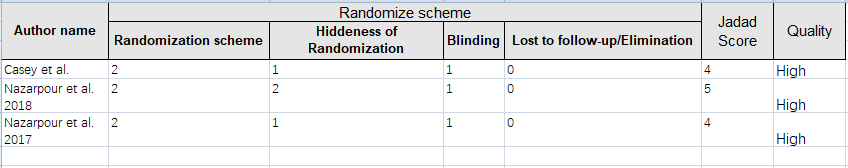


(B) Quality assessment - Jadad for RCT study. The score ranges from 0 to 5 and a total score of ≤3 or > 3 indicates low or high quality of reporting, respectively


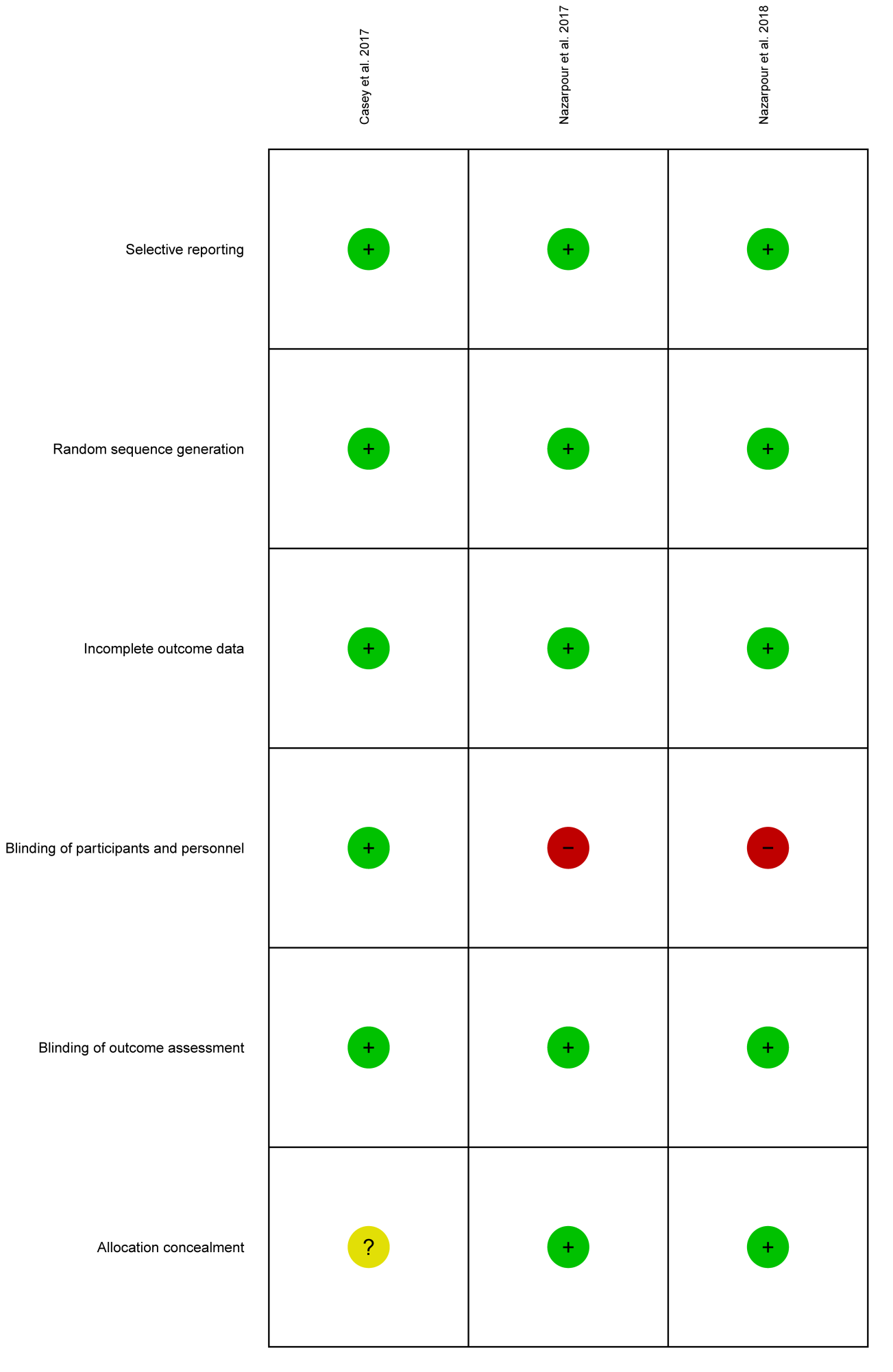


(C) Risk of bias assessment - RCT table


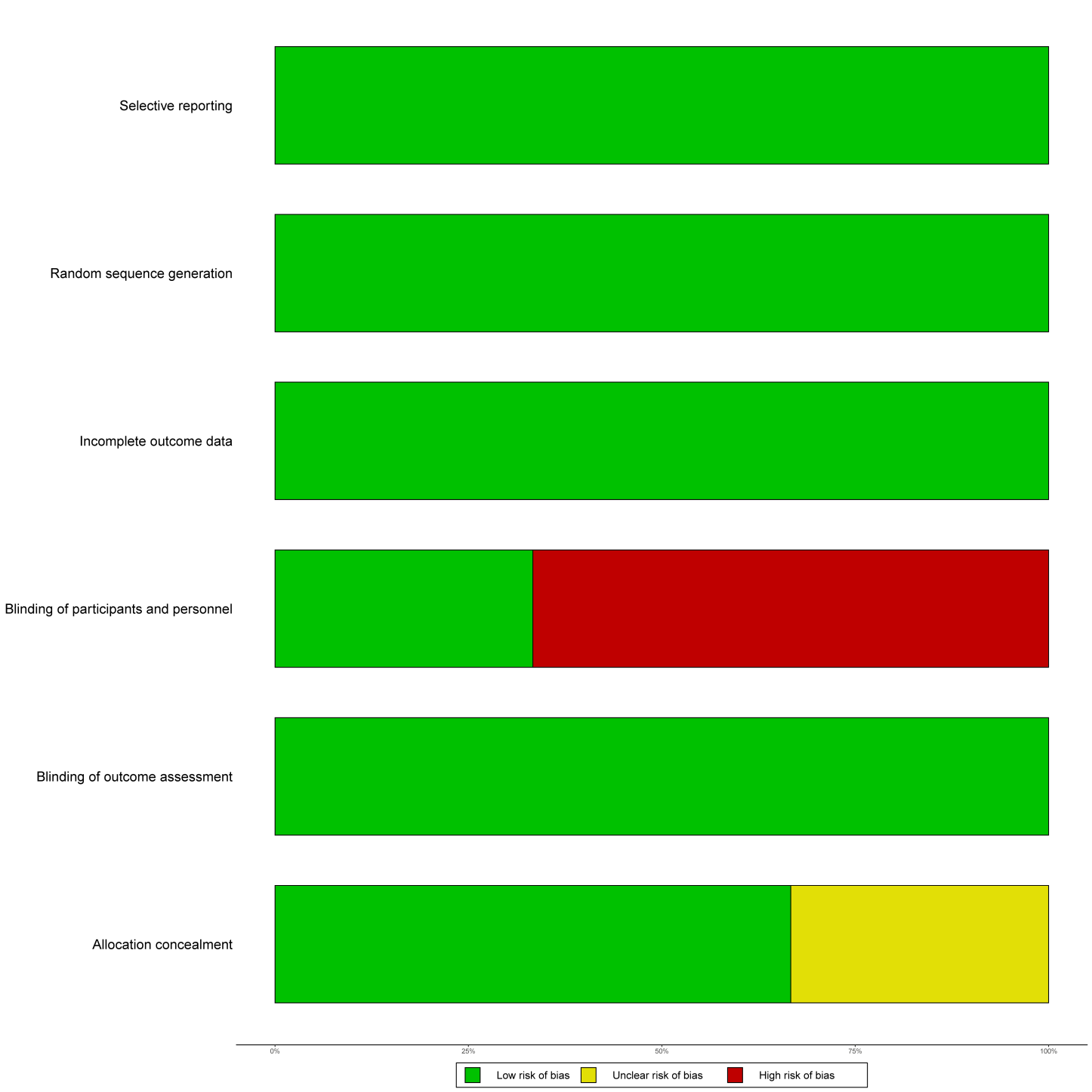


(D) Risk of bias assessment - RCT chart

**Supplementary Figure S2**: Forest plots of pooled results showed that there was not statistical evidence of an association between LT4 therapy and the risk of (A) gestational diabetes, (B) placental abruption, (C) fetal growth restriction or (D) small for gestational age


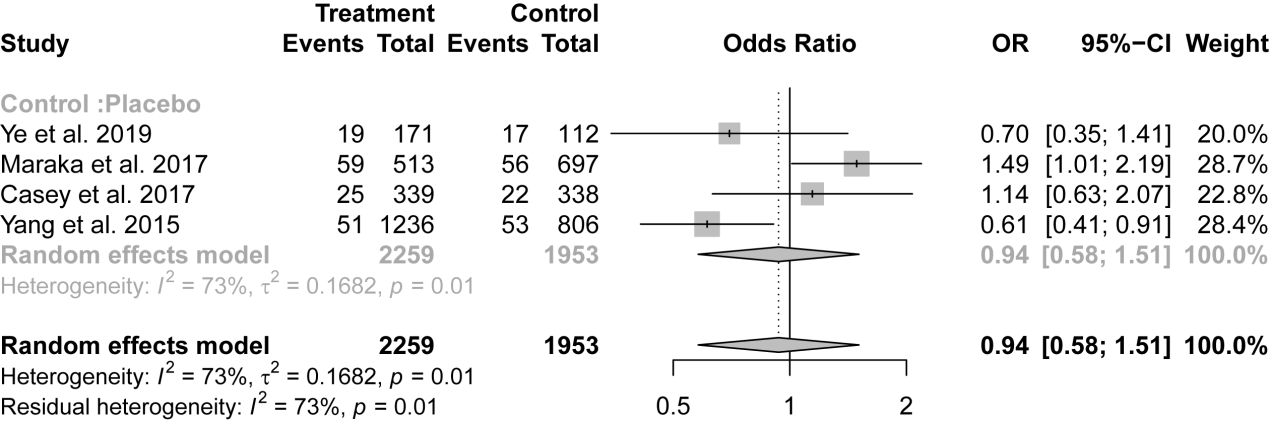


(A) Gestational diabetes


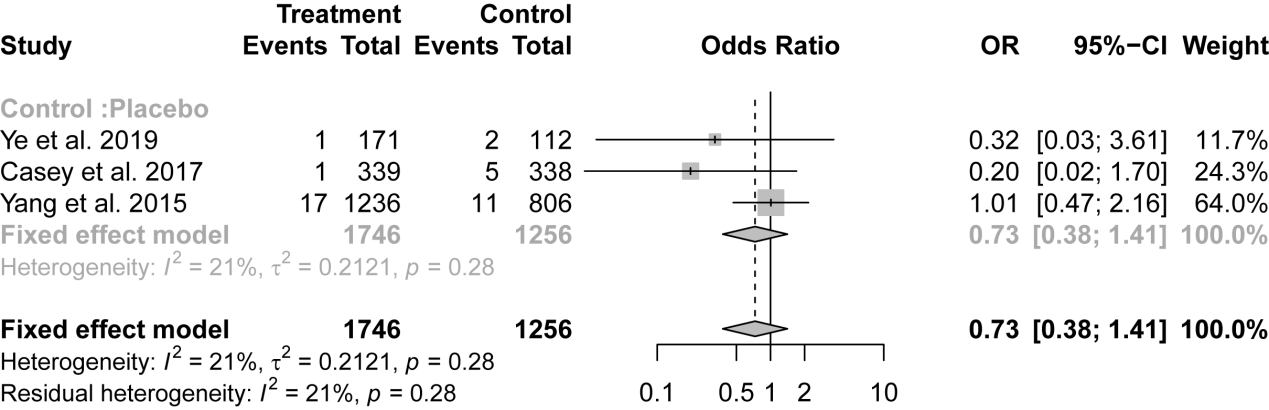


(B) Placental abruption


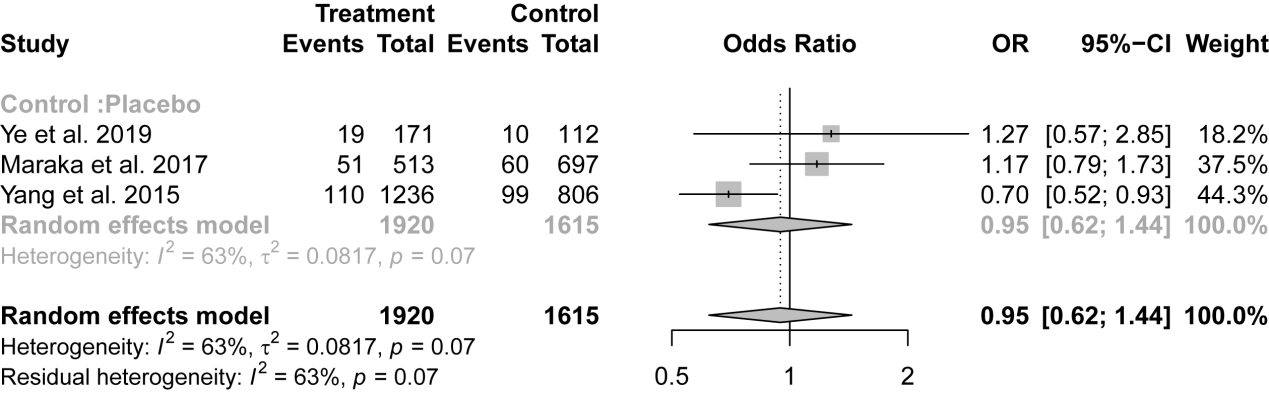


(C) Fetal growth restriction


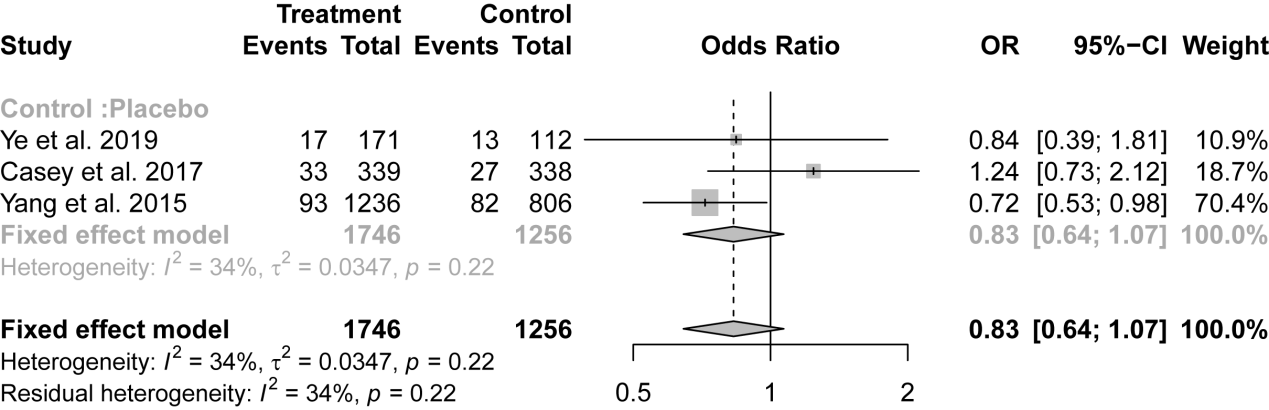


(D) Small for gestational age

**Supplementary Figure S3**: Sensitivity analysis. With regards to (A) preterm birth, the pooled ORs of the remaining studies were observed to change but no change was noted when removing Maraka et al. 2017 or Casey et al. 2017. With regards to (B) gestational hypertension, the pooled ORs of the remaining studies were observed to no change but change was noted when removing Yang et al. 2015. With regards to (C) pregnancy loss, after excluding individual studies, there were small changes in the pooled ORs but no change in the directionality of the effect and all the pooled ORs of the remaining studies were consistent with the overall effect

Omitting study OR 95%-CI p-value tau^2 I^2

Omitting Ye et al. 2019 0.6653 [0.4089; 1.0825] 0.1008 0.1753 64.5%

Omitting Nazarpour et al. 2018 0.6911 [0.4430; 1.0782] 0.1034 0.1436 61.5%

Omitting Maraka et al. 2017 0.5503 [0.3635; 0.8333] 0.0048 0.0869 41.9%

Omitting Nazarpour et al. 2017 0.7132 [0.4873; 1.0437] 0.0819 0.0931 52.1%

Omitting Casey et al. 2017 0.5600 [0.3165; 0.9908] 0.0464 0.2545 67.8%

Omitting Yang et al. 2015 0.5810 [0.3173; 1.0638] 0.0785 0.2933 67.9%

Pooled estimate 0.6323 [0.4098; 0.9756] 0.0383 0.1588 60.7%

(A) Preterm birth

Omitting study OR 95%-CI p-value tau^2 I^2

Omitting Ye et al. 2019 0.7875 [0.6352; 0.9763] 0.0293 0.0000 0.0%

Omitting Maraka et al. 2017 0.7745 [0.6142; 0.9767] 0.0308 0.0000 0.0%

Omitting Casey et al. 2017 0.7575 [0.5999; 0.9565] 0.0196 0.0000 0.0%

Omitting Yang et al. 2015 0.8407 [0.5979; 1.1821] 0.3184 0.0000 0.0%

Pooled estimate 0.7822 [0.6333; 0.9663] 0.0227 0.0000 0.0%

(B) Gestational hypertension

Omitting study OR 95%-CI p-value tau^2 I^2

Omitting Ye et al. 2019 0.5454 [0.4171; 0.7132] < 0.0001 0.0000 0.0%

Omitting Maraka et al. 2017 0.6585 [0.4652; 0.9322] 0.0185 0.0000 0.0%

Omitting Casey et al. 2017 0.5526 [0.4268; 0.7153] < 0.0001 0.0000 0.0%

Omitting Yang et al. 2015 0.4912 [0.3556; 0.6785] < 0.0001 0.0000 0.0%

Pooled estimate 0.5531 [0.4295; 0.7121] < 0.0001 0.0000 0.0%

(C) Pregnancy loss
